# Correction criteria for the qualitative analysis of the prison population: drugs possession/consumption and gender violence

**DOI:** 10.3389/fpsyt.2024.1413814

**Published:** 2024-08-01

**Authors:** Lucas Muñoz-López, Borja Fernández García-Valdecasas, Slava López-Rodríguez, Beatriz Aguilar-Yamuza

**Affiliations:** ^1^ Department of Personality, Evaluation and Psychological Treatment, Faculty of Education and Sports Sciences, University of Granada, Melilla, Spain; ^2^ Department of Theory and History of Education, Faculty of Education Sciences, University of Granada, Granada, Spain; ^3^ Department of Didactics of Language and Literature, University of Granada, Granada, Spain; ^4^ Department of Education, Faculty of Educational Sciences and Psychology, University of Córdoba, Córdoba, Spain

**Keywords:** qualitative analysis, language disorders, drugs possession or consumption, gender violence, writing

## Abstract

**Introduction:**

People with language difficulties cannot face challenges related to social skills. Those language disorders affect academic, work environments, and social interaction, leading to maladaptive and aggressive behaviors. Young inmates are at high risk of experiencing unrecognized language deficiencies. It is, therefore, necessary to analyze linguistic pathologies that can influence criminal behavior (drugs possession/consumption and gender violence crimes). There are many standardized tests to evaluate and detect language difficulties in adults in English. However, there are relatively few options in Spanish; there are no tests that evaluate language qualitatively and in depth. Most of the research is conducted with children and adolescents.

**Objectives:**

To propose a reliable coding system for the correction and interpretation of narratives (essays and narratives) from the Battery for the Evaluation of Writing Processes (PROESC) in the prisoners charged of drugs possession or consumption and gender violence crimes.

**Design:**

The sample was composed of 287 men.

**Main outcome measures:**

They completed the Demographic, Offense, and Behavioral Interview in Institutions, the International Personality Disorders Examination (IPDE), and PROESC.

**Results:**

We found that the proposed coding system presented high concordance, that is, high inter-rater reliability.

**Conclusion:**

The classification system for the correction and interpretation of narratives was shown to be reliable.

## Introduction

People with language difficulties cannot face challenges related to social skills. Fitzsimons and Clark (1) state that language disorders affect academic, work environments, and social interaction, leading to maladaptive and aggressive behaviors. Along the same lines, Morken et al. (2) highlight that young inmates are at high risk of experiencing unrecognized language deficiencies. It is, therefore, necessary to analyze linguistic pathologies that can influence criminal behavior (drugs possession/consumption and gender violence crimes).

Most standardized tests are currently focused on opaque languages such as English (2). However, there are barely any tests in Spanish that assess language in adults. In young adults, the Test de Evaluación de los Procesos de Escritura (PROESC; 3) evaluates the main processes involved in writing. It has adequate dictation tasks to assess each writing processing module and dictation tasks record the number of errors and the type of error made for later analysis the dictation tasks record the number of errors and the type of error made for later analysis. Besides, it allows comparison between comprehension processes in the two modalities of written language. In this way, it is possible to determine whether writing impairments are dependent on the written form or whether they involve a more generalized impairment process (Afonso et al., 2015; Carreteiro et al., 2016; 4, 5; Gutiérrez-Fresneda & Díez-Mediavilla, 2017; Gutiérrez-Fresneda, 2017; 6; Marques-de Oliveira et al., 2017; Martínez-García et al., 2019; 7; Nigro et al., 2015; 8).

This test constitutes a very structured evaluation procedure where the participant must respond according to the indications that appear at the beginning of the test and the instructions of the researcher. Paper and pencil tasks are inexpensive, flexible, and portable methods (9). However, while these tasks are very objective and easily replicable procedures, tasks 5 and 6 require an analysis qualitative.

Qualitative research fills a gap in the analysis of certain problems by adopting various content or discourse analysis procedures. The main objective of this technique is to describe the qualities of a phenomenon as a whole using a flexible approach. This technique begins from a holistic perspective, i.e., it tries to examine a specific situation in detail (10). It is based on the decomposition and classification of information collected through interviews, stories, observations, images, advertisements, news, and political discourse (11).

Qualitative aspects of language can also be evaluated, including the adequacy, precision, or magnitude of written expression. In some cases, it is possible to evaluate the ability to express the message correctly, often providing important additional information to help understand the written result (in the form of a narrative or essay). This type of study is mostly used in the prison population, particularly men convicted for drug offenses and violence (12). It has been shown that qualitative methodology is essential for studies with individuals belonging to these populations. Due to their characteristics and the type of experiences they present, this type of methodology allows for a deeper analysis, the results of which can inform the development of prevention and intervention processes.

Qualitative methodology uses a series of instruments that are not highly structured and standardized. Its scoring system is quite flexible, can be structured according to the objectives, and can be analyzed through qualitative procedures and transformed into quantitative data (13).

Qualitative aspects of language such as planning, transcription, and revision can also be evaluated. In some cases, it is possible to evaluate more specific aspects, such as decoding errors and informal aspects. The qualitative method comprises a series of instruments whose items are relatively unstructured and standardized, with a scoring system that can be used flexibly depending on the objectives. Moreover, the results can be analyzed through qualitative and quantitative procedures, transforming qualitative information into quantitative information. It is necessary to establish a coding system that corresponds to a model that can serve as a guide for analyzing and coding the writing.

Language difficulties in prisoners have attracted the attention of much of the scientific community for decades (1, 2). The authors highlight that there is a very diverse prevalence of writing disorders that may be due to the lack of consensus in the definition of dyslexia or reading-writing disorders. Due to the social nature of language, language in prisoners must be analyzed to enhance social inclusion. Morken et al. (2) points out that there is a relationship between the severity of a crime, the presence of an oral language disorder, and personality disorders. Therefore, the objective of this study was to propose a reliable coding system for the correction and interpretation of narratives and essays from the Writing Process Evaluation Battery (PROESC) (3) in the prison population.

## Participants

The sample consisted of 287 men mean age 37.69 (SD=8.84) from the Granada Penitentiary Center. The inclusion criteria were to have been charged of drugs possession or consumption and gender violence crimes. The exclusion criteria in both cases were being over 50 years, presenting a psychiatric illness (schizophrenia or depression), and receiving psychopharmacological treatment. First, participants were interviewed individually to check the inclusion criteria and, if eligible, were offered the opportunity to participate in the research. The interview was carried out by the prison psychologist and the duration of the interviews was not evaluated. They then took part in an individual session in which they completed the measures listed below. Participants were reminded at the beginning of the session of their right to discontinue the procedure at any time, and their written consent was then obtained. Once the data collection process was completed, the data were corrected. This study was approved by the Ethics Committee of the Autonomous Community of Andalusia (PEIBA, 0766-N-21).

## Procedure

Regarding the correction and interpretation of the narratives and essays, **Tables 1**, **2** were used for coding. Participants were requested to create two different writings, a narrative one about folk tale or story and a free topic essay. The speech-language pathologist conducted the task. The analysis of the narratives and essays were developed by three evaluators (speech-language pathologist, linguist, and expert in quality and care management). To calculate the inter-rater reliability, three evaluators coded the narratives and essays. **Table 3** presents a proposal of correction criteria obtained considering **Tables 1**, **2**.

**Table 1:** Findings of the analyzed studies.

Authors	Motive	Findings	References
**Benítez, 2000**	Aspects to be evaluated in the generation of texts	Elements to be evaluated in texts: organizational criteria	Benítez-Figari, R. (2000). The rhetorical situation: Its importance in learning and teaching written production. *Signos Journal*, *33*(48), 49-67. https://dx.doi.org/10.4067/S0718-09342000004800005. https://dx.doi.org/10.4067/S0718-09342000004800005
**Bereiter and Scardamalia (1987)**	Basic text with elements of writing processes	Psychological processes in writing	Bereiter C. & Scardamalia M. (1987). Fostering Reflective Process. In The Psychology of written composition (389). New York: Routledge.
**Berninger et al. (1994)**	Contributions to writing levels	Intraindividual differences in writing levels (syllables, words, phrases, sentences, paragraphs, texts)	Berninger, W.V., Mizokawa, D.T., Bragg, R., Cartwright A. & Yates, C. (1994) Intraindividual Differences in Levels of Written Language. *Reading & Writing Quarterly*, 10:3, 259-275, https://doi.org/10.1080/1057356940100307
**Berninger et al. (2015)**	Aspects to be evaluated in written texts	Sub-words (handwriting), words (spelling) and syntax (sentence composition)	Berninger, V. W., Nagy, W., Tanimoto, S., Thompson, R., & Abbott, R. D. (2015). Computer instruction in handwriting, spelling, and composing for students with specific learning disabilities in grades 4-9. *Computers and Education.* https://doi.org/10.1016/j.compedu.2014.10.005
**Berninger et al. (2008)**	Previous research on dyslexia and its environment	Previous studies have focused on reading, not writing.	Berninger, V. W., Nielsen, K. H., Abbott, R. D., Wijsman, E., & Raskind, W. (2008). Writing problems in developmental dyslexia. *Journal of School Psychology*, 46 (2008) 1-21 Writing. https://doi.org/10.1016/j.jsp.2006.11.008
**Etchepareborda et al. (2001)**	Neuroanatomical basis of dyslexia	Early studies on the brain and dyslexia	Etchepareborda, M., Etchepareborda, M., & Habib, M. (2001). Neurobiological Basis of Phonological Awareness: Compromise of This. *Dyslexia*. 5-23.
**Graham (1999)**	Basic characteristics of dyslexia	Writing difficulties can interfere with the performance of other composition processes and restrict writing development	Graham, S. (1999). Handwriting and spelling instruction for students with learning disabilities: A review. *Learning Disability Quarterly*. 22(2), 78-98. https://doi.org/10.2307/1511268
**Graham (1999)**	Metawriting	The influence of spelling errors on perceptions of writing ability. Difficulties in literacy affect the rate of writing and the course of writing development.	Graham, S. (1999). The role of text production skills in writing development: A special issue - I. *Learning Disability Quarterly*. 22(2), 75-77. https://doi.org/10.2307/1511267
**Hayes and Flower (1980)**	Aspects to evaluate in the generation of texts	Elements to evaluate in texts: planning, translation, and proofreading	Hayes, J. R., & Flower, L. (1980). Identifying the Organization of Writing Processes. In L. W. Gregg, & E. R. Steinberg (Eds.), Cognitive Processes in Writing: An Interdisciplinary Approach (pp. 3-30). Hillsdale, NJ: Lawrence Erlbaum.
**Herrada-Valverde and Herrada-Valverde (2018).**	Adult writing models	Writing skills of adults with difficulties in producing texts.	Herrada-Valverde, G, & Herrada-Valverde, R. I. (2018). Procedural competencies to elaborate written summaries: the case of students of the Faculty of Education of the University of Salamanca. *Mexican Journal of Educational Research*, *23*(77), 505-525. Retrieved June 05, 2021, from http://www.scielo.org.mx/scielo.php?script=sci_arttext&pid=S1405-66662018000200505&lng=es&tlng=es.
**Kellogg and Raulerson (2007)**	Specific aspects of proofreading	Elements to be evaluated in texts: correct spelling, punctuation, etc.	Kellogg, R.T., Raulerson, B.A. (2007). Improving the writing skills of college students. *Psychonomic Bulletin & Review.* 14, 237-242. https://doi.org/10.3758/BF03194058.
**Longcamp et al. (2016).**	Contribution of the neuroscience of writing	Handwriting processes in adults with handwriting difficulties.	Longcamp, M., Richards, T. L., Velay, J. L., & Berninger, V. W. (2016). Neuroanatomy of Handwriting and Related Reading and Writing Skills in Adults and Children with and without Learning Disabilities: French-American Connections. Pratiques, 171-172, 3175. https://doi.org/10.4000/pratiques.3175.
**Richards et al. (2017)**	Neuroimaging in writing	Writing tasks and instructions during neuroimaging tests: DTI, fMRI	Richards, T. L., Berninger, V. W., Yagle, K. J., Abbott, R. D., & Peterson, D. J. (2017). Changes in DTI Diffusivity and fMRI Connectivity Cluster Coefficients for Students with and without Specific Learning Disabilities In Written Language: Brain’s Response to Writing Instruction. *Journal of Nature And Science*, *3*(4), e350. Available in PMID: 28670621; PMCID: PMC5488805
**Rincón-Camacho (2013)**	Describe writing and learning	Processes related to the generation and planning of texts	Rincón-Camacho, L. J. (2013). Los estilos cognitivos: una aproximación al estudio de las diferencias individuales en la composición escrita: An approach to the study of individual differences in written composition. *Revista Colombiana de Educación*, (64), (64), 107-130. https://doi.org/10.17227/01203916.64rce107.130
**Singer and Bashir (2004)**	General aspects of proofreading	Elements to evaluate in texts: planning, generation, revision, and organization of texts.	Singer, Bonnie & Bashir, Anthony (2004). Developmental Variations in Writing Composition Skills. In A.Stone, E.R. Silliman, B.J. Ehren & K. Akpel (Eds.), Handbook of Language & Literacy. The Guilford Press: New York.
**Tanimoto et al. (2015).**	Characteristics of the population with dyslexia	Major difficulties in handwriting, spelling, morphology and phonetics, comprehension, and composition.	Tanimoto, S., Thompson, R., Berninger, V. W., Nagy, W., & Abbott, R. D. (2015). Computerized Writing and Reading Instruction for Students in Grades 4 to 9 With Specific Learning Disabilities Affecting Written Language. *Journal of Computer Assisted Learning*, *31*(6), 671-689. https://doi.org/10.1111/jcal.12110.
**Thompson et al. (2016)**	Specific aspects of proofreading	Elements to be evaluated in texts: spelling, among others.	Thompson, R., Tanimoto, S., Berninger, V., & Nagy, W. (2016). Coding, reading, and writing: Integrated instruction in written language. 2016 IEEE Symposium on Visual Languages and Human-Centric Computing (VL/HCC), 73-77. https://doi.org/10.1109/VLHCC.2016.7739667.
**Wallis et al. (2017).**	General aspects of proofreading	Elements to be evaluated in texts: transcription and text generation	Wallis, P., Richards, T., Boord, P., Abbott, R., & Berninger, V. (2017). Relationships between Translation and Transcription Processes during fMRI Connectivity Scanning and Coded Translation and Transcription in Writing Products after Scanning in Children with and without Transcription Disabilities. *Creative education*, *8*(5), 716-748. https://doi.org/10.4236/ce.2017.85055

**Table 2:** Text correction criteria from the reviewed literature categorized according to Gutiérrez-Fresneda (2018).

Categories	Processes	Definition	Author’s correction	Self-correction
**PLANNING** **Preverbal representation**	Generation	Retrieval of words or segments that facilitate the creation of a theme (Hayes and Flower, 1980).	**Hayes and Flower (1980):** - Recovery using memory - Potentially useful recovered items - Evaluation of recovered elements - Writing notes	- Recovery using memory - Potentially useful recovered items - Analyzes the recovered elements - Write notes
	Organization		**Benítez et al. (2000):** - Topic selection - Relate the task to the objective of the evaluation. - Define the scope of rhetorical purposes. - Write the task in a clear way - Evaluate the quality of the subject **Hayes and Flower (1980):** - Usefulness of the subject - Identify, if possible, the first or the last topic. - Order and respect topics according to order of appearance - Search for data to stay on topic - Identify category	- Select a topic - Define the scope of the purposes of the text - Write the task clearly - Evaluates the quality of the subject - Identifies the first or last topic of the text - Order and respect topics according to order of appearance - Relates data to stay on topic
	Establishment of goals	Selection of what is generated in the “Generation” process.		
**TRANSLATION**		Transform into text, from memory, following the planning guide (Hayes and Flower, 1980).	**Kellogg and Raulerson (2007)):** - Correct spelling - Scoring - Grammar - Diction (correct use of words) - Thematic sentences - Main idea - Consistent links **Singer and Bashir (2004):** - Phonological awareness - Morphosyntax - Appropriate semantics - Cohesion and consistency **Hayes and Flower (1980):** - Good form - Full text - Grammatically correct sentences - Logical structure - Structured paragraphs	Words: - Correct spelling - Score - Grammar (morphosyntax) - Diction (correct use of words) - Thematic sentences - Main idea present - Consistent links Text: - Good form - Full text - Logical structure - Structured paragraphs - Appropriate semantics
**REVIEW. Perception and self-correction**	Reading	Examine written material (Hayes and Flower, 1980).		
	Editing	Detect and correct possible errors in the previous processes (Hayes and Flower, 1980).	**Hayes and Flower (1980):** - Spelling errors - Grammar errors - Search for alternatives - Word errors - Elimination of ambiguities - Change to common words - Uniformity	- Detection and correction of spelling errors - Detection and correction of grammar errors - Search for alternatives - Word error detection and correction - Detection and correction of ambiguity errors - Change to common words

**Table 3:** PROESC correction proposal: Text writing (narrative and essay).

CATEGORIES	VARIABLES	DEFINITION
**WORDS AND PARAGRAPHS**	Number of words	This consists of counting the total number of words in the text.
	Number of paragraphs	The aim is to check the organization within the text Paragraphs into which the text could have been divided. For example, this will be scored according to the missing paragraphs.
**ERRORS RELATING TO** **FORMAL ASPECTS**	Number of punctuation errors	This involves checking for punctuation, i.e., the use of periods, commas, exclamations, question marks, and hyphens.
	Number of lines not respecting margins	Refers to the framing of the text on the page, such as tabs, margins, and enumerations.
	Number of incorrect separations between words	We look for fragmented words or broken words. Example: un fortunately, ha bí a
	Number of incorrect word conjunctions	The number of incorrect conjunctions between words that appear throughout the text is computed. The aim is to look for the phenomenon of coarticulation, i.e., the joining of words. Example: habersi, demiabuela.
	Number of repetitions	The appearance of two consecutive occurrences of the same complete word is counted. Example: On the, I went to my mother’s house). Emphasis of an affirmation or negation is not considered repetition. Example: porque me gusta porque si, es guapo guapo. Number of incorrect repetitions
	Number of words with unreadable handwriting	Words that cannot be read because of alteration of grapho-motor aspects (when the writing stroke is so altered that it is not possible to distinguish the letters to identify the word) are computed.
	TOTAL	TOTAL (Sum of the items of the Errors Relating to Formal Aspects)
**DECODING ERRORS**	Number of Substitutions	Refers to the substitution of one letter/grapheme for another. For example, pallaso for payaso, empello for empeño, olo for ola, lla instead of ya.
	Number of Additions	Refers to whether a letter/grapheme is added. For example, addictions for additions, haver si instead of a ver si, Hera instead of era.
	Number of Omissions	Refers to whether a letter/grapheme is removed. Example: ola for hello, sensibiidad for sensibilidad, tre instead of tres.
	Number of Inversions	This refers to the change of order of the letter/grapheme, consonant, or vowel. For example, Plalta instead of plata, honor instead of horno, Lavaro instead of Alvaro.
	Number of Rotations	This is the writing of a letter/grapheme in mirror image. Letters can also be rotated on their own axis. Example: pombo instead of bombo; agua for ana.
	Number of Lexicalizations	Indicates changing a complete word for another, e.g., minister for marriage, active for perspective.
	Number of incorrect accents	Indicates misplaced accents, either accents that are not where they should be or accents that are where they should not be. For example, jamon instead of jamón or jámon instead of jamón.
	TOTAL	TOTAL (Sum of the Decoding Errors items)
**GRAMMAR**	Number of grammatically incorrect sentences	Indicates the number of grammatically misspelled sentences with either an incorrect preposition, a misconjugated verb, or incorrect gender-number agreement.
**MAIN AND SECONDARY IDEAS**	Appearance of the main idea	This refers to whether the main idea can be found easily when reading the text, that is, what is being talked about (daily routine, story of Little Red Riding Hood). This is why it is important to take into account the title.
	Appearance of secondary ideas	This refers to whether we can find secondary ideas that enrich the text.
**PLANNING ERRORS**	Number of disconnections between the main idea and the title	Number of times that the main idea is unrelated to the title of the text. Number of times an idea unrelated to the main idea of the text appears.
	Number of times that secondary ideas do not appear	Number of times that secondary ideas do not appear and should appear. Number of times the common thread (plot) is lost. Refers to additional information. For example, in the stories, what Little Red Riding Hood carries in her basket and how many push-ups she does per day when describing her daily routine.
	Number of deviations from thematic continuity	This refers to the number of times that events do not follow a sequence (thread). For example, In the morning I exercise, I get up and have breakfast and then I eat but in the middle of the morning I go the pottery workshop.
	Number of times technical vocabulary not used	This refers to the non-use of specific words related to the text. If talking about the mechanical aspects of cars, the tools will be mentioned, and the name of the tools would be classified as technical vocabulary. Or if talking about a physical activity, it is important to specify what kind of activities are performed, for example, squats and sit-ups would be regarded as technical vocabulary. Thus, “I get up in the morning and exercise my tummy” should have instead read “ I do sit-ups”.
	Number of times coherent vocabulary not used	Words that do not fit in with the theme of the text, that is, presence of words that have nothing to do with the subject of the text. For example, when talking about a forest, the writer should refer to pine trees, and when talking about physical exercise, they should refer to abs.
	Number of times varied vocabulary not used	Repeats the same word several times in the same sentence and does not use synonyms and/or antonyms. For example, my car was really cool, we had really cool races and got really cool tattoos.
	TOTAL	TOTAL (Sum of the Planning Errors items)
**VOCABULARY**	Use of technical vocabulary	This refers to the use of specific words related to the text. Example: if talking about the mechanical aspects of cars, the tools should be mentioned, and the names of the tools would be regarded as technical vocabulary. Or when talking about a physical activity, the writer should specify what kind of activities are performed; for example, squats and sit-ups would be regarded as technical vocabulary.
	Use of coherent vocabulary	This refers to the use of words whose meaning is in accordance with the text. For example, when talking about a forest, reference is made to pine trees, and when talking about physical exercise, referring to abs.
	Use of varied vocabulary	This refers to the use of a wide variety of words, including use of synonyms and antonyms.
	TOTAL	TOTAL (Sum of Vocabulary items)
**REVISION**	Number of modifications made to the text	This checks whether the user corrects letters, words, or group of words. The correction is scored according to whether an error is identified, corrected, and made visible in the text. A score is given according scored whether the correction has been done well. For example, a crossed-out word next to the new word or proposal.

## Instruments

### Demographic, crime, and institutional behavior interview

This interview was designed for this research study and consists of collecting information about sociodemographic data, type of offenses (drug possession and/or consumption and gender violence crimes) and their penalties, and sanctions within the prison according to the Prison Regulations (Royal Decree 1201/1981, May 8, Articles 107 and 108).

### Writing Processes Evaluation Battery (PROESC)

This is an individual test that aims to evaluate the main processes involved in creating texts. It is composed of six tests, which are: 1) Syllable dictation; 2) Word dictation; 3) Pseudoword dictation; 4) Sentence dictation; 5) Writing a narrative and 6) Writing an essay. In this study, we used tests 5 and 6, which assess the ability to plan a narrative and an expository text. Although the instrument (3) has a high internal consistency of 0.82 (alpha coefficient) in the first four tests, it lacks quantitative criteria for the correction and interpretation of the writing tests (5 and 6). For this reason, in this study, we used only tasks 5 and 6.

## Data analysis

Data analyses were conducted using the SPSS Statistics 22.0 program. The analysis of inter-rater concordance was performed by calculating the kappa index and Pearson correlations to address.

## Results

### Inter-rater reliability analysis

Regarding inter-rater reliability, the concordance analysis yielded very high coefficients (see **Table 4**).

**Table 4:** Inter-rater reliability [Kappa (K) and Pearson (P) coefficients].

CATEGORIES	VARIABLES	NARRATIVES						ESSAYS					
		E1-E2		E2-E3		E1-E3		E1-E2		E2-E3		E1-E3	
		K	P	K	P	K	P	K	P	K	P	K	P
**WORDS AND PARAGRAPHS**	Number of words	0.09	0.99	0.46	0.99	0.09	0.99	0.10	0.98	0.75	0.99	0.10	0.97
	Number of paragraphs	0.95	0.99	0.89	0.99	0.93	0.99	0.48	0.80	0.85	0.89	0.55	0.80
**ERRORS RELATING TO** **FORMAL ASPECTS**	Number of punctuation errors	1	1	0.93	1	0.93	0.99	0.98	1	0.93	0.99	0.95	0.99
	Number of lines not respecting margins	1	1	0.98	1	0.98	1	1	1	1	1	1	1
	Number of incorrect separations between words	0.52	0.89	0.79	0.91	0.56	0.85	1	1	1	1	1	1
	Number of incorrect word conjunctions	0.89	0.99	0.84	0.99	0.93	0.99	1	1	1	1	1	1
	Number of repetitions	1	1	1	1	1	1	1	1	1	1	1	1
	Number of words with unreadable handwriting	1	1	1	1	1	1	1	1	1	1	1	1
	TOTAL	0.82	0.99	0.84	0.99	0.77	0.99	0.98	1	0.93	0.99	0.95	0.99
**DECODING ERRORS**	Number of Substitutions	0.16	0.90	0.38	0.92	0.43	0.96	0.23	0.87	0.84	0.98	0.18	0.87
	Number of Additions	0.99	0.99	0.95	0.99	0.97	0.99	0.97	0.99	0.94	0.99	0.97	0.99
	Number of Omissions	0.96	0.99	0.95	0.99	0.98	0.99	0.36	0.89	0.89	0.99	0.38	0.89
	Number of Inversions	0.41	0.73	0.64	0.84	0.42	0.75	0.74	0.85	0.70	0.92	0.76	0.86
	Number of Rotations	NOT GIVEN						0.80	0.27	NOT GIVEN			
	Number of Lexicalizations	1	1	1	1	1	1	0.95	0.96	0.94	0.99	0.89	0.95
	Number of incorrect accents	0.93	0.99	0.87	0.99	0.93	0.99	0.91	0.99	0.87	0.99	0.96	0.99
	TOTAL	0.98	1	0.96	1	0.98	1	1	1	1	1	1	1
**GRAMMAR**	Number of grammatically incorrect sentences	0.50	0.96	0.41	0.93	0.40	0.95	0.96	0.99	0.93	0.99	0.97	0.99
**MAIN AND SECONDARY IDEAS**	Appearance of the main idea	1	1	1	1	1	1	1	1	1	1	1	1
	Appearance of secondary ideas	0.89	0.99	0.81	0.99	0.91	0.99	0.47	0.97	0.47	0.94	0.53	0.96
**PLANNING ERRORS**	Number of disconnections between the main idea and the title	0.99	0.99	0.98	0.99	0.99	0.99	1	1	1	1	1	1
	Number of times that secondary ideas do not appear	0.87	0.99	0.87	0.99	1	1	1	1	1	1	1	1
	Number of deviations from thematic continuity	1	1	1	1	1	1	1	1	1	1	1	1
	Number of times technical vocabulary not used	0.98	0.99	0.97	0.99	0.99	0.99	1	1	1	1	1	1
	Number of times coherent vocabulary not used	1	1	1	1	1	1	1	1	1	1	1	1
	Number of times varied vocabulary not used	0.99	1	0.99	1	1	1	1	1	1	1	1	1
	TOTAL	0.97	0.99	0.95	0.99	0.98	0.99	1	1	1	1	1	1
**VOCABULARY**	Use of technical vocabulary	1	1	0.96	0.99	0.96	0.99	0.99	1	0.95	0.99	0.95	0.99
	Use of coherent vocabulary	1	1	1	1	1	1	1	1	1	1	1	1
	Use of varied vocabulary	1	1	1	1	1	1	0.98	0.99	0.98	0.99	0.99	1
	TOTAL	1	1	0.96	0.99	0.96	0.99	0.98	1	0.93	0.99	0.94	1
**REVISION**	Number of modifications made to the text	1	1	1	1	1	1	1	1	1	1	1	1
**NET TOTAL**		0.1	0.99	0.4	1	0.1	0.99	0.04	0.98	0.59	0.99	0.04	0.97

## Discussion

Analyzing language difficulties in the prison population, charged of drugs possession or consumption and gender violence crimes, through writings (narratives and essays) may be relevant to discover specific issues and identifying the differences in this population. For this reason, and according to the reviewed bibliography (**Tables 1**, **2**), we have proposed a categorization system for the interpretation of the writings of the prison population.

This study aimed to provide a reliable coding system for correcting and interpreting narratives and essays from the Writing Process Evaluation Battery (PROESC) (3). We found that the proposed coding system presented high concordance, that is, high inter-rater reliability. Furthermore, the degree of agreement was very high for all the proposed categories. This classification provides novel and useful information for the evaluation of writing processes. Furthermore, the context in which this study has been conducted — a prison setting — advances our understanding of the writing difficulties of inmates that have, until now, never been analyzed. The results obtained are in line with Busetto et al. (10)Douglass et al. (13) and Moser and Korstjens (11), who point out the importance of creating, developing and applying qualitative evaluation methods to develop more detailed means of analysis and gain in-depth knowledge of the samples received from participants in various studies. In our study, we can verify that the categorization created from the PROESC (3) could conscientiously show the possible alterations in language and writing that prison population could suffer.

According to Larrazabal et al. (9), the use of classical or traditional means is very useful and reliable to know in detail the language alterations of the inmate population. To this we add the analysis created from the categorization proposed in this study to obtain a very reliable and viable evaluation method (10). Therefore, this study is the first to propose a model for categorizing and correcting texts in both narratives and essays while confirming its reliability and effectiveness through a comprehensive inter-rater analysis.

## Conclusions

There are few studies where language in prisoners is analyzed. This is why we highlight the novel nature of this study, since it proposes a model for categorization and correction of texts, both narratives and essays, which exhaustively study their reliability and effectiveness through interjudge analysis. To identify the difficulty of writing in the prison population that have used and trafficked with drugs, or have committed gender violence crimes, the following categories should be considered: Words and Paragraphs, Errors Related to Formal Aspects, Decoding Errors, Grammar, Revision and Net Total, Main and Secondary Ideas, Vocabulary, Planning Errors, Words and Paragraphs, Errors Related to Formal Aspects, Decoding Errors.

Although individuals know phoneme-grapheme correspondence rules, language disturbances of a reiterative and persistent nature may appear in those who show aggressive behavior (those participants who committed gender violence or drugs trafficking and/or consumption crimes). This finding could be related to co-occurrences in the behavior of compulsive individuals and those with learning difficulties. Language therapy in patients with high levels of compulsivity could improve self-control and self-criticism, thereby enhancing the capacity to form social relationships and show empathy.

Knowing the linguistic skills of this part of society is vital to know in detail social aspects of prisoners. Furthermore, given that the main reason for incarceration is to work on social inclusion, we must know the state of this social stratum. Since the job of penitentiaries is to reintroduce inmates and make them proactive elements in society, we must rehabilitate all altered aspects of them. This is why we must develop useful tools to know the linguistic status and knowledge of prisoners so that they can fully access the language, enhance their social inclusion and achieve their job placement. After having carried out this analysis and having delved into the existing studies, new questions arise: why are there no studies that analyze language disorders in the prison population? Why are there no language tests for adults? Why are there no language tests for adults? Is there no qualitative method to analyze language?

We have detected several limitations in our study. Our sample has been reduced to men with a series of crimes determined to evaluate language. This is because the number of women in the penitentiary center was small and the majority did not meet the inclusion criteria, so they were discarded. Our future lines of work will focus on analyzing the female prison population. On the other hand, although the results of the interjudge analysis are positive, another limitation found is having a low, although representative, number of evaluators.

## Data availability statement

The datasets presented in this study can be found in online repositories. The names of the repository/repositories and accession number(s) can be found below: https://digibug.ugr.es/handle/10481/89488.

## Ethics statement

Participants were reminded at the beginning of the session of their right to discontinue the procedure at any time, and their written consent was then obtained. Once the data collection process was completed, the data were corrected. This study was approved by the Ethics Committee of the Autonomous Community of Andalusia (PEIBA, 0766-N-21).

## Author contributions

LM: Writing – review & editing, Writing – original draft. BF: Writing – review & editing, Writing – original draft. SL: Writing – review & editing, Writing – original draft. BA-A: Writing – review & editing, Writing – original draft.

## References

[1] Fitzsimons D Clark A. Pausing mid-sentence: an ecological model approach to language disorder and lived experience of young male offenders. Int J Environ Res Public Health. (2021) 18:1225. doi: 10.3390/ijerph18031225 33573031 PMC7908202

[2] Morken F Jones LØ. Helland WA. Disorders of language and literacy in the prison population: A scoping review. Educ Sci. (2021) 11:77. doi: 10.3390/educsci11020077

[3] Cuetos F Ramos JL Ruano E. PROESC. In: Evaluación de los procesos de escritura. TEA, Madrid (2004).

[4] Davies R Rodríguez-Ferreiro J Suárez P Cuetos F. Lexical and sub-lexical effects on accuracy, reaction time and response duration: Impaired and typical word and pseudoword reading in a transparent orthography. Reading Writing. (2013) 26:721–38. doi: 10.1007/s11145-012-9388-1

[5] Guarnieri-Mendes G Domingos-Barrera S. Phonological processing and reading and writing skills in literacy. Paideía. (2017) 27:298–305. doi: 10.1590/1982-43272768201707

[6] Jiménez J,E García E Venegas E. Are phonological processes the same or different in low literacy adults and children with or without reading disabilities? Reading Writing. (2010) 23:1–18. doi: 10.1007/s11145-008-9146-6

[7] Megino-Elvira L Martín-Lobo P Vergara-Moragues E. Influence of eye movements, auditory perception, and phonemic awareness in the reading process. J Educ Res. (2016) 109:567–73. doi: 10.1080/00220671.2014.994197

[8] Rodríguez-Pérez C González-Castro P Álvarez L Álvarez D Fernández-Cueli M. Neuropsychological analysis of the difficulties in dyslexia through sensory fusion. Int J Clin Health Psychol. (2012) 12:69–80.

[9] Larrazabal AJ García Cena CE Martínez CE. Video-oculography eye tracking towards clinical applications: A review. Comput Biol Med. (2019) 108:57–66. doi: 10.1016/j.compbiomed.2019.03.025 31003180

[10] Busetto L Wick W Gumbinger C. How to use and assess qualitative research methods. Neurological Res Pract. (2020) 2:1–10. doi: 10.1186/s42466-020-00059-z PMC7650082 33324920

[11] Moser A Korstjens I. Series: Practical guidance to qualitative research. Part 1: Introduction. Eur J Gen Pract. (2017) 23:271–3. doi: 10.1080/13814788.2017.1375093 PMC8816396 29185831

[12] Heith C Beaton B Ayeni D Dabney D Tewksbury R. A content analysis of qualitative research published in top criminology and criminal justice journals from 2010 to 2019. Am J Criminal Justice. (2020) 45:1060–79. doi: 10.1007/s12103-020-09540-6

[13] Douglass JE Constantino C Alvarado J Verrastro K Smith K. Qualitative investigation of the speech-language therapy experiences of individuals who covertly stutter. J Fluency Disord. (2019) 61:105713. doi: 10.1016/j.jfludis.2019.105713 31451301

